# Viral Infection and Autophagy Dysregulation: The Case of HHV-6, EBV and KSHV

**DOI:** 10.3390/cells9122624

**Published:** 2020-12-07

**Authors:** Maria Anele Romeo, Roberta Santarelli, Maria Saveria Gilardini Montani, Roberta Gonnella, Rossella Benedetti, Alberto Faggioni, Mara Cirone

**Affiliations:** 1 Department of Experimental Medicine, Sapienza University of Rome, 00161 Rome, Italy; mariaanele.romeo@uniroma1.it (M.A.R.); roberta.santarelli@uniroma1.it (R.S.); mariasaveria.gilardinimontani@uniroma1.it (M.S.G.M.); roberta.gonnella@uniroma1.it (R.G.); benedetti.1589832@studenti.uniroma1.it (R.B.); alberto.faggioni@uniroma1.it (A.F.); 2 Laboratory Affiliated to Istituto Pasteur Italia-Fondazione Cenci Bolognetti, 00161 Rome, Italy

**Keywords:** HHV-6, EBV, KSHV, autophagy, UPR, ROS, DCs, AD, cancer

## Abstract

Human Herpes Virus-6 (HHV-6), Epstein-Barr Virus (EBV) and Kaposi Sarcoma Herpes Virus (KSHV) are viruses that share with other member of the Herpesvirus family the capacity to interfere with the autophagic process. In this paper, mainly based on the findings of our laboratory, we describe how, through different mechanisms, these viruses converge in reducing autophagy to impair DC immune function and how, by infecting and dysregulating autophagy in different cell types, they promote the pathologies associated with their infection, from the neurodegenerative diseases such Alzheimer’s disease to cancer.

## 1. HHV-6, EBV and KSHV and Associated Diseases

Human Herpes Virus-6 (HHV-6), Epstein-Barr Virus (EBV) and Kaposi Sarcoma Herpes Virus (KSHV) belong to the Herpesvirus family, even if the first is a betahepesvirus, while the EBV and KSHV are gammaherpesviruses. All members of this family are characterized by the possibility of establishing two types of infections: Latent infection, in which only a minority of genes are expressed, and; lytic infection, in which all viral genes are expressed and viral replication occurs. These viruses infect several cellular types although the efficiency and the type of infection may be different among them. HHV-6 comprises two different viruses sharing more than 80% homology, namely HHV-6A and HHV-6B, both isolated for the first time from immune-suppressed patients [1], but are characterized by a different capacity to infect target cells [2]. HHV-6B is the causative agent of exanthema subitum, a self-limiting disease that arises in young children [3], while no diseases have been shown to be induced by HHV-6A primary infection. Both viruses are neurotropic and have been associated with Central nervous system (CNS) diseases such multiple sclerosis (MS) and Alzheimer’s disease (AD). EBV and KSHV are instead oncoviruses strongly associated with human B cell lymphomas: Primary Effusion Lymphomas (PEL), in the case of KSHV and Burkitt’s Lymphoma, lymphoproliferative diseases typical of immune-compromised patients and about 40% of Hodgkin Lymphoma in the case of EBV. Besides hematological cancers, KSHV is present and plays a role in the pathogenesis of all forms of Kaposi’s Sarcoma, while EBV is strongly linked to nasopharyngeal carcinoma [4] and to some subtypes of gastric cancers [5]. As for other herpesviruses, HHV-6, EBV and KSHV are able to impair immune response, which is not surprising when considering that those viruses are able to persist lifelong in the infected hosts. For this purpose, in previous studies, we found that these viruses may interfere with the monocyte differentiation into dendritic cells (DCs) [6,7,8,9], preventing the formation of cells that play a pivotal role in initiating and regulating immune responses, including those against cancer [10,11] and viruses [12].

## 2. Autophagy Regulation and Dysregulation

Autophagy is a catabolic process required for the maintenance of cellular homeostasis and to adapt to stressful conditions [13]. It is also strongly involved in the anti-microbial immune response. Autophagy comprises three processes, namely macroautophagy, usually referred as autophagy, microautophagy and chaperon-mediated autophagy (CMA). Autophagy is regulated by a group of autophagy-related (ATG) proteins that control all its steps, from autophagosome formation to the degradation of the autophagic cargo and autophagosome membranes into the lysosomes [13]. The initiation of the autophagic process is controlled by several molecular pathways, the most important of which are Target of Rapamycin (mTOR) and 5′ Adenosine Monophosphate-activated Protein Kinase (AMPK). They sense the availability of nutrients and are able to detect other types of stress such as those caused by hypoxia and Reactive Oxygen Species (ROS) accumulation. Autophagy can be either non-selective or selective. In the latter case, it mediates the degradation of specific cargo, i.e., mitochondria (mitophagy), endoplasmic reticulum (reticulophagy) or invading microrganisms (xenophagy), leading, in such cases, to the direct elimination of intracellular microbes, including viruses through the lysosomal route. In addition, autophagy contributes to antiviral response being involved in MHC-class II and class I-mediated antigen presentation [14], and being required for DC formation from monocyte precursors [15]. That said, it can be quite expected that viruses try to subvert autophagy to avoid their own elimination and to persist in the infected host. However, besides this, viruses may, in some cases, take advantage of the autophagosome formation and utilize these vesicles for their intracellular transport during their replication, as observed in the case of EBV and KSHV that promote the first autophagic steps, while blocking the last ones [16,17].

A proper functioning of autophagy is required not only for immune response, as autophagy helps to prevent accumulation of ROS and unfolded proteins, regulating the activation of pro-survival pathways such as Mitogen-Activated Protein Kinases (MAPKs), Signal Transducer and Activator of Transcription 3 (STAT3) and Nuclear Factor-κB (NF-kB), as well as the release of pro-inflammatory cytokines [18]. Also, because the reduction of autophagy may increase Endoplasmic Reticulum (ER) stress and contribute to the triggering of the unfolded protein response (UPR), it plays a key role in the inflammatory process. While, inflammation is in principle, a defensive process, when not properly controlled, it may predispose to several diseases, including autoimmune diseases and cancer [19]. In the latter case, the major role seems to be played by the dysregulation of the selective autophagy called mitophagy, which leads to the accumulation of damaged mitochondria that are the main source of ROS. As a consequence of autophagy reduction the accumulation of p62 occurs, as this molecule is mainly degraded through autophagy. Its role in cancerogenesis is quite controversial, as it may, either promote tumorigenesis by activating NF- κB and induce apoptosis by activating caspase 8 or even stabilize NRF2, a molecule having a contradictory role in cancer [20]. Autophagy dysregulation, ER stress and inflammation are also involved in CNS diseases, not only the autoimmune ones, i.e., multiple sclerosis (MS), but also neurodegenerative diseases such as Alzheimer’s disease (AD) [21] and Parkinson’s Disease (PD) [22].

## 3. HHV-6, Autophagy Dysregulation and Pathologies

In previous studies, we have highlighted that infection by HHV-6B derived from exanthema subitum patients dysregulated autophagy in monocytes, increased ER stress and activated UPR as a strategy to impair the in vitro differentiation of monocytes into DCs [9]. Even if the induction of this immunosuppressive effect by HHV-6 infection was previously reported [23], our study shed more light into the mechanisms responsible for it. Another consequence of autophagy reduction and UPR dysregulation caused by HHV-6B infection in monocytes was the increase of intracellular ROS and the inter-connected activation of STAT3 and STAT1 pathways, responsible for the up-regulation of the PD-L1 surface expression [24]. PD-L1 is an immune checkpoint inhibitor whose expression on cells presenting antigens to T lymphocytes induces the exhaustion of the latters and thus causes immune dysfunction [25].

Interestingly, as we recently showed, autophagy reduction and UPR dysregulation also occurs in astrocytes and primary neurons infected by HHV-6A. In this case, such effects promoted beta-amyloid (Aβ) intracellular and extracellular accumulation as well as tau protein hyper-phosphorylation, phenomena that characterize AD together with neuroinflammation [24]. Furthermore, inflammatory cytokines are released following HHV-6 infection of monocytes [9] and, as these cells can cross the brain-blood barrier and contribute to the formation of microglia, their infection could play a role also in neuroinflammation. In such study, we found that the activation of the Protein kinase R-like Endoplasmic Reticulum Kinase (PERK) branch of UPR was involved in HHV-6A-induced protein tau hyper-phosphorylation while the Inositol-Requiring protein-1α (IRE1 alpha) and Activating Transcription Factor 6 (AFT6) arms of UPR played a minor role. This is in agreement with other previous studies reporting that PERK may activate Glycogen Synthase Kinase-3 (GSK-3) beta, a kinase known to directly phosphorylate tau protein [26]. It has been recently reported that HHV-6 A and HHV-6 B may play a role in cancerogenesis, as it can stimulate cell growth and block apoptosis, interfere with epigenetic regulation and cooperate with oncogenic viruses such as EBV, HPV and KSHV. It will be interesting to evaluate if autophagy dysregulation by HHV-6A and/or B could occur in cells from which tumor arise and if this mechanism could contribute to their-induced pro-tumorigenic effects [27].

EBV, the first human oncovirus discovered, is able to block the last autophagic steps when its replication is induced by opportune stimuli in lymphoma cells harboring viral infection in a latent state. This is not very surprising as autophagic vesicles that may contain viral particles end up in the lysosomes where their content is degraded [16]. Moreover, in this study, as well as in studies from other’s laboratories, EBV has been shown to exploit the autophagic machinery to enhance viral production [28]. However, other herpesviruses such as Varicella Zoster (VZV) have been shown to allow a complete autophagic flux during the activation of their lytic cycle as they can resist the degradative activity of lysosomal proteases [29]. Although, other studies indicated that this virus can also block the last autophagic steps [30]. Dysregulating the activation of pathways involved in the autophagy induction, such as PKR/EIF2 alpha and mTOR, is another strategy put in place by other viruses belonging to the Herpesvirus family, such as Herpes Simplex virus-1 (HSV-1) or cytomegalovirus (HCMV) [31]. The latter, similarly to EBV, is able to promote the initial autophagic phases and inhibit the last ones, as its protein TRS1 interact with Beclin 1 [32]. As for HHV-6, EBV infection also reduces autophagy in infected monocytes, leading to p62/SQSTM1 accumulation and Nuclear factor erythroid 2-Related Factor 2 (NRF2) up-regulation, preventing ROS increase induced by Granulocyte-Macrophage Colony-Stimulating Factor (GM-CSF) and Interleukin-4 (IL-4). As ROS is one of the main drivers of monocyte differentiation into DCs, their reduction strongly impaired the formation of these cells. Moreover, EBV reduced mitochondrial biogenesis in monocytes and through this mechanism it further prevented ROS production [8]. Interfering with DC formation represents an important strategy to escape from immune recognition, together with the other immune suppressive strategies that EBV is able to put in place [33].

It is well-established that a proper functioning of autophagy is required for cancer prevention, especially because its selective form, the mitophagy, is responsible for the elimination of damaged mitochondria that produce ROS [34]. Being EBV an oncogenic virus, it is able to reduce autophagy in B cells, the major targets of EBV infection, to facilitate their oncogenic transformation [35]. The accumulation of ROS, the activation of STAT3 pathway and Interleukin-6 (IL-6) secretion, effects previously shown to be involved in EBV-driven LCL immortalization [36], were indeed exacerbated by autophagy reduction [9]. Several proteins belonging to EBV have been shown to affect autophagy and among them there are some expressed during the latency, such as LMP1 [37] and EBNA3 [38] and other expressed during lytic cycle activation such as BFRF1 [39].

## 4. KSHV, Autophagy Dysregulation and Pathologies

Differently from HHV-6A, HHV-6B and EBV, KSHV is not an ubiquitous herpesvirus. Its infection, with the exception of some geographical areas, occurs indeed in only 5% of general population. During the activation of its lytic cycle in PEL cells, KSHV, similarly to EBV, blocks the last autophagic steps by down-regulating Ras-related protein (RAB) 7, which regulates lysosome biogenesis and the fusion of autophagosomes with lysosomes [17]. The inhibition of monocyte differentiation is a consequence also of KSHV infection [40], even if we have previously shown that KSHV-infected lymphoma cells secrete a variety of cytokines that may per se contribute to the impairment of DC differentiation and function [6]. Also, in this case, we have demonstrated that the reduction of autophagy was the main mechanism involved in this virus-induced immune suppressive effect, although the mechanism leading to it was quite different. Indeed, KSHV dysregulated the balance between calpains and calpastatin, reducing the latter and leading to cleavage of ATG5 [7]. As said above, KSHV is a human oncovirus. HUVEC cells can be used as a model to study its-mediated oncogenic in vitro transformation. These endothelial cells indeed, following KSHV infection, change their phenotype becoming spindle cells that closely resemble Kaposi’s Sarcoma cells. Knowing the role of autophagy in preventing cancer onset and the capacity of KSHV to reduce it in other cell types, we focused on the impact of KSHV infection on autophagic process to evaluate whether autophagy dysregulation could be involved in HUVEC transformation. As we recently showed, mTOR activation by the KSHV, effect previously demonstrated in other studies [41], was responsible for autophagy reduction in endothelial cells, promoting endothelial to mesenchymal transition (EndMT), triggering UPR and promoting the production of pro-inflammatory cytokines [19]. Besides through modulating pathways affecting autophagy, several KSHV proteins can directly influence this process, as for example v-cyclin and v-Flip [42].

## 5. Future Perspective

**Figure 1:**
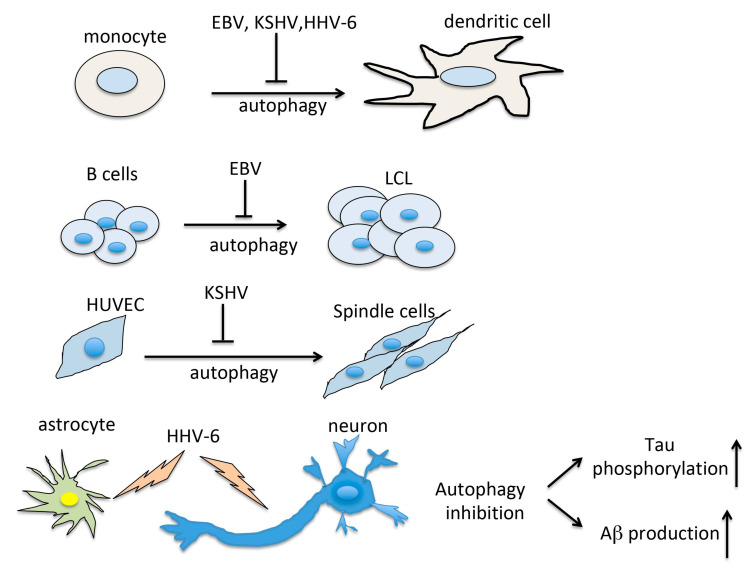
Scheme representing the consequence of autophagy inhibition by HHV-6, EBV and KSHV.

In conclusion, based on the above reported studies, it emerges that autophagy reduction contribute to immune dysfunction and promote the onset of disease associated with EBV, KSHV and HHV-6, as summarized in Figure 1. Moreover, as a consequence of autophagy reduction, an accumulation of p62/SQSTM1 occurs, as this multifunctioning protein is mainly degraded through a complete autophagic flux [13]. As it has been observed that p62 accumulated during the in vitro immortalization of B cells into LCL and during HUVEC transformation into spindle cells [9,19], studies are in progress in our laboratory to assess the role of p62 in the carcinogenesis driven by these viruses. This molecule indeed, among its numerous functions, has been shown to regulate DNA Damage Response (DDR) that strongly influences cancer onset and progression [43]. p62 also contributes to shape the tumor microenvironment that has a strong impact on immune response, regulating for example M1/M2 balance and affects fibroblast trans-differentiation into Cancer Associated Fibroblasts (CAF) [44]. Interestingly, the skewing of macrophages towards M2 differentiation is another effect that can be directly induced by KSHV infection of macrophages [45] and is possible that EBV might do so either. However, in vivo studies are required to better clarify whether the manipulation of autophagy, and whether UPR could be an effective strategy to improve the overall outcome of organism survival in the course of herpesvirus infection.
